# Association between physical frailty and cortical structure in middle-aged and elderly people: a Mendelian randomization study

**DOI:** 10.3389/fnagi.2024.1395553

**Published:** 2024-05-22

**Authors:** Xin Zhang, Zhen Wang, Jing Zou, Le Zhang, Jing-Hua Ning, Bei Jiang, Yi Liang, Yu-Zhe Zhang

**Affiliations:** ^1^College of Basic Medical Sciences, Dali University, Dali, Yunnan, China; ^2^The First Affiliated Hospital of Dali University, Dali, Yunnan, China; ^3^Yunnan Key Laboratory of Screening and Research on Anti-pathogenic Plant Resources from West Yunnan (Cultivation), Dali, Yunnan, China; ^4^Princess Margaret Cancer Centre, TMDT-MaRS Centre, University Health Network, Toronto, ON, Canada

**Keywords:** physical frailty, cerebral cortical structure, Mendelian randomization, middle-aged and older adults, aging

## Abstract

**Introduction:**

Physical weakness is associated with cortical structures, but the exact causes remain to be investigated. Therefore, we utilized Mendelian randomization (MR) analysis to uncover the underlying connection between frailty and cortical structures.

**Methods:**

The Genome-Wide Association Study (GWAS) on frailty pooled data from publicly available sources such as the UK Biobank and included five indicators of frailty: weakness, walking speed, weight loss, physical activity, and exhaustion. GWAS data on cerebral cortical structure were obtained from the ENIGMA consortium, and we assessed the causal relationship between hereditary frailty and cortical surface area (SA) or cortical thickness (TH). Inverse variance weighting (IVW) was used as the primary estimate, and heterogeneity and multidimensionality were monitored by MR-PRESSO to detect outliers. Additionally, MR-Egger, Cochran’s Q test, and weighted median were employed.

**Results:**

At the aggregate level, there was no causal relationship between frailty and cortical thickness or surface area. At the regional level, frailty was associated with the thickness of the middle temporal lobe, parahippocampus, rostral middle frontal lobe, lower parietal lobe, anterior cingulate gyrus, upper temporal lobe, lateral orbital frontal cortex, pericardial surface area, rostral middle frontal lobe, upper temporal lobe, rostral anterior cingulate gyrus, lower parietal lobe, and upper parietal lobe. These results were nominally significant, and sensitivity analyses did not detect any multidirectionality or heterogeneity, suggesting that the results of our analyses are reliable.

**Discussion:**

The results of our analyses suggest a potential causal relationship between somatic weakness and multiple regions of cortical structure. However, the specific mechanisms of influence remain to be investigated. Preliminary results from our analysis suggest that the effects of physical frailty on cortical structures are influenced by various factors related to frailty exposure. This relationship has been documented, and it is therefore both feasible and meaningful to build on existing research to explore the clinical significance of the relationship.

## 1 Introduction

As the aging of the population increases, physical frailty is also a growing concern. The gradual weakening of the functional reserves of various physiological systems in middle-aged and older adults leads to frailty, which results in reduced adaptability to the surrounding environment (including, for example, reduced resistance to climate change, pathogenic microorganisms, etc.) and increased vulnerability of the body (Clegg et al., 2013). Conceptualized as a multifactorial medical syndrome, frailty is mainly characterized by a decrease in endurance, physiological functions, etc., which leads to an increased risk of disease occurrence and prognosis, and a weakening of the body’s resistance to unfavorable factors in the surrounding environment (Morley et al., 2013). Therefore, frailty is actively advocated in clinical practice as a routine monitoring program to prevent its adverse consequences.

The occurrence of frailty and its complications poses a significant challenge for public health, clinical diagnosis, and treatment (Hoogendijk et al., 2019). Studies have shown that the onset and progression of frailty may be attributed to the ongoing deterioration of brain reserve function (Chen et al., 2015; Del Brutto et al., 2017; Zijlmans et al., 2021). For example, the hallmark of brain aging is an increase in the total white matter density and gray matter volume of the brain - the implications of which for frailty are clear (Chen et al., 2015; Kant et al., 2018). We know that the vast majority of psychiatric disorders in older adults are associated with neurodegenerative changes, i.e., changes in brain structure. However, fewer studies have been conducted on the relationship between frailty and cortical structure, and there are no uniform conclusions. Therefore, it is urgent to understand the association between frailty and brain structure (Genon et al., 2022; Marek et al., 2022). The current Mendelian randomization analysis method is uniquely suited for studying a variety of undiscovered potential causal relationships. Therefore, the use of MR to explore the link between frailty and cortical brain structure is of interest. Similarly, this is important for the preliminary analysis of psychiatric disorders that occur due to altered cortical structure.

Mendelian randomization (MR) is a method used to predict associations between etiology and disease by utilizing genetic variants with strong correlations with exposure as instrumental variables. This approach serves as an effective solution to the bias introduced by macroscopic observations (Emdin et al., 2017). Mendelian randomization is currently widely used in studies of brain structure and Alzheimer’s disease, migraine, and alcohol consumption (Mavromatis et al., 2022; Guo et al., 2023; Seyedsalehi et al., 2023). Unfortunately, no studies on frailty have been reported. Therefore, we designed a systematic MR analysis based on a prospective study on the association between frailty and cortical structure in UK Biobank (Jiang et al., 2023). The aim was to understand the potential association between frailty and cortical structure and to preliminarily analyze the impact of altered cortical structure on psychiatric disorders in middle-aged and older adults.

## 2 Materials and methods

### 2.1 Source of data on physical frailty and brain structure

The GWAS data for physical frailty were statistically analyzed by UK Biobank^1^ for a UK population with frailty indicators between 2006 and 2010. Jiang et al. (2023) reported detailed information on the participants, including weakness (*N* = 719,433), walk speed (*N* = 358,974), weight loss (*N* = 355,129), exhaustion (*N* = 350,580), and physical activity (*N* = 539,757) (Jiang et al., 2023). GWAS data related to cortical structure were obtained from the ENIGMA Consortium (Grasby et al., 2020),^2^ where 51,665 participants from around the world (comprising 60 cohorts, with the European population accounting for more than 94% of the total) underwent MRI scans to assess cerebral surface area (SA) and cerebral cortical thickness (TH). An approximate partition of the cerebral cortex was established for the two average regions of the cerebral hemispheres. Additionally, an approximate anatomical partition was based on the depth between the sulci, using the 34 regions defined by the Desikan-Killiany atlas of brain structure (Desikan et al., 2006). They conducted independent measurements and GWAS analyses of both total surface area (SA) and total thickness (TH) of the cerebral cortex, as well as SA and TH of the 34 regions. In the GWAS analysis, SA and TH at the global level were used as covariates. Meta-analysis was performed using METAL when the data were all completed with quality control (Willer et al., 2010). Our study utilized the findings from the meta-analysis conducted on the European population (Supplementary Material b, Supplementary Table 1 for comprehensive cohort details).

### 2.2 Physical frailty-related phenotypes

According to the description of Fried’s frailty phenotype (Fried et al., 2001), frailty mainly consists of five indicators: weakness, walk speed, exhaustion, weight loss, and physical activity. The severity of frailty is related to the number of frailty phenotypes that a person possesses. In general, individuals are considered frail when they exhibit three or more of the aforementioned indicators of frailty (Zhu et al., 2023). As there is no broad consensus on the definition of frailty, we relied on the prevailing definition of frailty in the existing literature to align with the GWAS data on frailty in UK Biobank (Supplementary Material a).

### 2.3 Selection of instrumental variables (IV)

**FIGURE 1:**
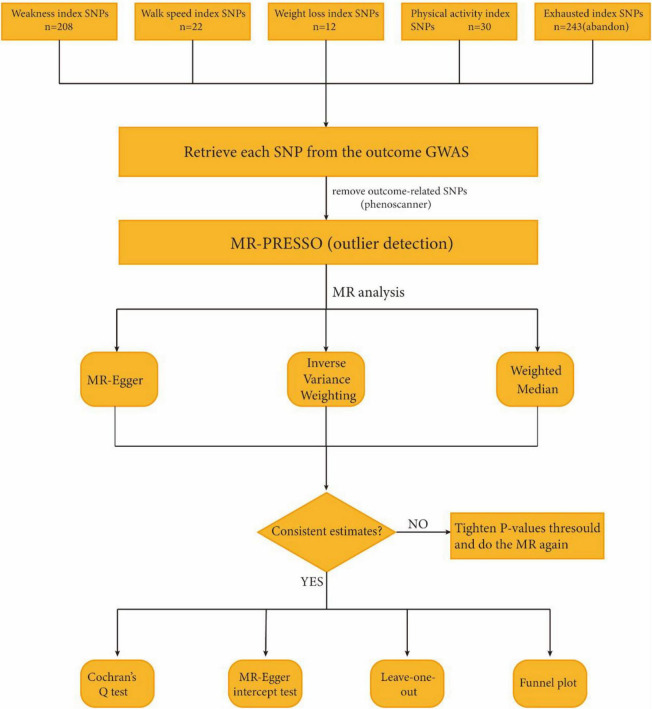
Study flame chart of the Mendelian randomization study to reveal the causal relationship between physical frailty and the brain cortical structure.

Single nucleotide polymorphisms (SNPs) loci with significant *P*-values were screened for frailty GWAS data based on the classification of Fried vulnerability phenotypes, including weakness and walk speed (*P* < 5.0 × 10^–8^), as well as weight loss and physical activity (*P* < 5.0 × 10^–6^). To ensure the validity of instrumental variables (IVs), it was necessary to establish a strong correlation with the exposure factor without introducing bias. The linkage disequilibrium condition was set to (LD): r2 < 0.001, and the clump window size was set to 1000 kb. SNPs that exhibited allelic incongruence with the exposure factor, as well as those with duplications or potential effects on the outcome, were excluded. Additionally, MR outliers (MR-) test and multivariate analysis (MVA) were conducted. The MR-PRESSO test and pleiotropy residual (MR Pleiotropy RESidual) were applied to assess potential horizontal pleiotropy. To eliminate the effect of pleiotropy, outliers were removed (Verbanck et al., 2018). The flowchart of the study is shown in Figure 1.

### 2.4 Mendelian randomization analysis

We conducted a two-sample Mendelian randomization (MR) analysis to examine the causal relationship between exposure factors and outcomes. We employed three different MR analysis methods, namely random effects inverse variance weighted (IVW), MR-Egger, and weighted median, to assess this relationship. Three assumptions need to be met for instrumental variable (IV): (1) the correlation assumption: the instrumental variable is strongly correlated with the exposure variable; (2) the independence assumption: the instrumental variable and the confounders are independent of each other; and (3) the exclusivity assumption: the effect of the instrumental variable on the outcome is mediated through the exposure variable. Therefore, even if only one genetic variant is null, the final result will be biased (Burgess et al., 2013). IVW was then used as the primary outcome of the analysis. In addition, MR-Egger and weighted median were employed to enhance the completeness of the results obtained from IVW and provide a more compelling extension to the broader range of applications for IVW (Chen et al., 2021). MR-Egger is used to detect heterogeneity and the presence of pleiotropy in the analysis. The presence of pleiotropy is indicated when the MR-Egger intercept deviates from 0 (Bowden et al., 2015). In addition, the weighted median test produces consistent estimates when weights from valid IVs are ≥50% because it uses median IVs (Bowden et al., 2016). To assess the stability of the causal relationship between exposure and outcome, sensitivity analyses were conducted on the results. These analyses included the MR-Egger intercept test and leave-one-out analysis. The purpose of these analyses was to further evaluate horizontal pleiotropy and to verify that individual SNPs did not have a significant impact on the obtained results. In addition, funnel plots were used to assess pleiotropy. Cochran’s Q test was used to identify heterogeneity, which was considered significant when Cochran’s Q (*P* < 0.05). In addition, the F statistic was used to estimate weak variable bias by calculating F. Instrumental variables with *F*-values less than 10 indicate weak bias and should be discarded (Burgess and Thompson, 2011). The formula for F is as follows: F = R^2^(N-2)/(1-R^2^), where R^2^ = (2 × eaf × (1-eaf) × beta^2^)/[(2 × eaf × (1-eaf) × beta^2^) + (2 × eaf × (1-eaf) × N × se^2^)]. In this formula, N represents the sample size, eaf is the effect allele frequency, beta is the genetic effect, and se is the standard error of the genetic effect (Papadimitriou et al., 2020).

To determine whether SNPs were associated with potential risk factors [mainly smoking (Corley et al., 2019), alcohol consumption (Mavromatis et al., 2022), educational level (Cox et al., 2016), blood pressure (Newby et al., 2022), mental illness (Gaser et al., 2004), etc.], we also searched for gene-phenotype associations provided by PhenoScanner^3^ and removed this portion of SNPs that might affect the outcome of SNPs (Staley et al., 2016; Chen et al., 2021).

### 2.5 Data analysis

All analyses were performed using the TwoSampleMR package (version 0.5.7) in R (version 4.3.1) and MR-PRESSO (version 1.0). In analyses at the level of the brain as a whole, a causal relationship between exposure and outcome was considered significant at *P* < 0.05. A total of 690 MR estimates were generated at the regional level for each cortical structure region, and the Bonferroni-corrected *P*-value should be 0.05/690. However, a result with *P* < 0.05 is not considered a negative result; instead, it is considered nominally significant (Chen et al., 2021).

### 2.6 Ethics

The data used in this study were derived from publicly available data obtained from studies of participants (from the ENIGMA Consortium and UK Biobank, respectively), which have been approved by the Ethics Committee for human experimental data and do not require separate ethical approval.

## 3 Results

### 3.1 Selection of instrumental variables

After a series of screening steps, we selected a total of 527 SNPs for predicting the outcome. These included 208 SNPs predicting frailty (*P* < 5 × 10^–8^) (Supplementary Material b, Supplementary Tables 2, 3), 22 SNPs predicting walking speed (*P* < 5 × 10^–8^) (Supplementary Material b, Supplementary Table 4), 243 SNPs predicting fatigue (*P* < 5 × 10^–5^) (Supplementary Material b, Supplementary Table 4), 12 weight loss SNPs (*P* < 5 × 10^–6^) (Supplementary Material b, Supplementary Table 5), 42 physical activity SNPs (*P* < 5 × 10^–6^) (Supplementary Material b, Supplementary Table 5), and 12 weight loss SNPs (*P* < 5 × 10^–6^) (Supplementary Material b, Supplementary Table 5). Individuals (Supplementary Material b, Supplementary Tables 6, 7). The independent variable (IV) was analyzed using the F-statistic data structure >10, indicating that there was no significant bias in the selected variables. In addition, neither the MR-Egger nor MR-PRESSO global tests detected any evidence of multiple effects. Finally, after passing the MR-PRESSO outlier test, a randomized analysis of association between multiple SNPs containing the four exposure factors and the results was subsequently performed using three different MR methods.

### 3.2 Relationship between exposure factors and outcomes

**FIGURE 2:**
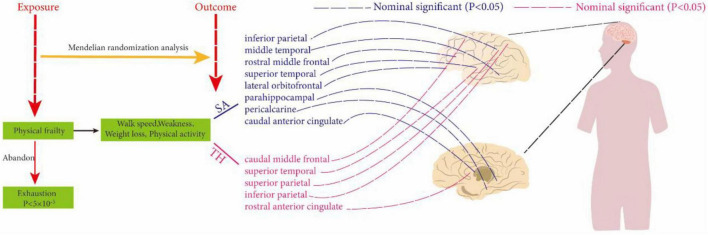
Using two-sample Mendelian randomization analysis to reveal the relationship between physical frailty and cerebral cortical structure. The dotted line indicates a non-significant positive result.

By conducting MR analysis, no significant causal relationships were found at the global level between weakness, walk speed, weight loss, physical activity, and SA and TH of cortical structures. However, at the regional level, our analysis revealed that walk speed was associated with the caudal anterior cingulate without weighted SA (IVW: β = 75.1694 mm, 95% CI = 21.2146 mm to 129.1247 mm, *P* = 0.006321). Defaults to IVW results without special annotation); weight loss versus lateral orbitofrontal (IVW: β = −152.156 mm, 95% CI = −266.812 mm to −38.2976 mm, *P* = 0.008872); weakness versus parahippocampal (IVW: β = −14.957 mm, 95% CI = −26.1247 mm, *P* = 0.006321) casts doubt on the causal relationship between these three key exposure factors and altered cerebral cortical structure as the most significant results in our analyses. The weighted median and MR-Egger also demonstrated the relationship between each exposure factor’s physical frailty association with cortical structure. Not only that, exposure factors related to physical frailty were found to be associated with various brain regions, including the caudal middle temporal, superior temporal, rostral middle frontal, rostral anterior cingulate, inferior parietal, and pericalcarine, as well as the superior parietal and other interconnected regions. Although the strength of these associations may not be very strong, they may still hold some meaningful implications (Supplementary Material b, Supplementary Table 8) and (Figure 2).

### 3.3 Sensitivity analysis

**FIGURE 3:**
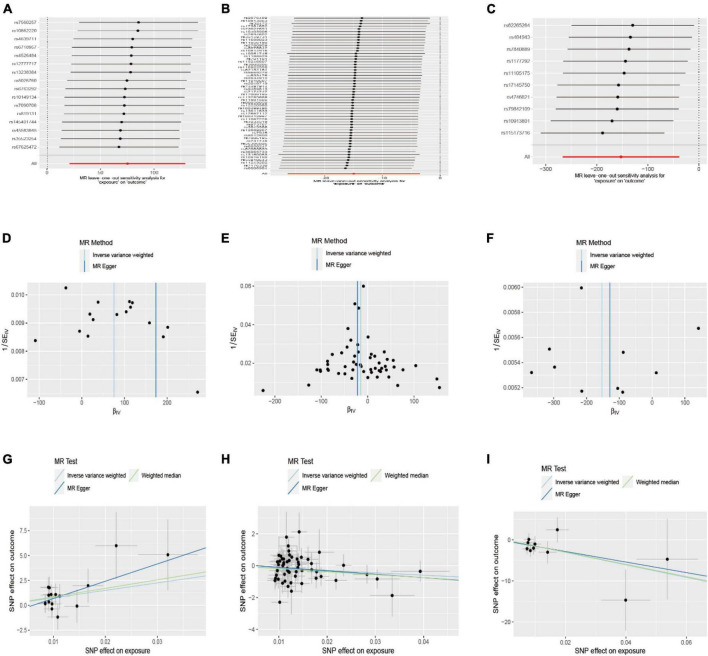
Leave-one-out and funnel plot of the significant Mendelian randomization statistics. **(A,D,G)** Association between walk speed and caudal anterior cingulate without global; **(B,E,H)** association between weakness and parahippocampal; **(C,F,I)** association between weight loss and lateral orbitofrontal.

Sensitivity analyses were conducted to assess the stability of the nominally significant results. These analyses included Cochran’s Q test, MR-PRESSO global test, leave-one-out analysis, funnel plot, and MR-Egger intercept test. All *p*-values >0.05 for MR-Egger, and the leave-one-out analysis showed no evidence of horizontal pleiotropy. Funnel plot symmetry on both sides showed that the estimates were not affected by individual SNPs, suggesting that there was no directional pleiotropy in the estimates that were unaffected by individual SNPs and biased (Figure 3). Cochran’s Q test showed that all *p*-values were greater than 0.05, indicating that the results were not heterogeneous. Additionally, the MR-PRESSO analysis did not identify any outliers or evidence of heterogeneity.

## 4 Discussion

With the aging of the body, physiological functions continue to weaken, resulting in a decreased ability to withstand various risk factors from pathogenic factors in the environment, such as climate change and impacts from pathogenic organisms (Fried, 2016). This frailty is particularly pronounced in middle-aged and elderly individuals, especially those over the age of 75–80 years. The relationship between frailty and the structure of the cerebral cortex originated from a prospective study by Jiang et al. (2023) based on UK Biobank data on frailty, health outcomes, and brain structure. Their study revealed a broad spectrum of connections between frailty and health outcomes, as well as brain structure, in middle-aged and older adults. However, it did not establish a direct causal relationship between frailty and gray matter in the brain. Only 50% of brain regions exhibited a negative correlation between gray matter volume and the severity of frailty. However, subcortical regions showed a strong correlation with frailty. Therefore, based on the prospective study of frailty and cortical structure, we assessed the potential causal relationship between frailty and cortical structure. To the best of our knowledge, based on our review of the relevant research literature, this study represents the first comprehensive analysis of frailty and cortical structure of the brain on a large scale. After making necessary corrections, our study reveals a correlation between frailty and cortical structure. Although we did not find any statistically significant positive results, the nominally positive results could still provide valuable insights in the fields of public health and clinical practice.

Overall, although our study did not find exposure factors with strong correlations on brain structure, non-significant causality is not negligible. According to our results, the effects of physical weakness on various brain regions are widespread, mainly on the middle temporal, caudal middle frontal, parahippocampal, rostral middle frontal, and rostral anterior cingulate, inferior parietal, caudal anterior cingulate, superior temporal, lateral orbitofrontal, pericalcarine, and superior parietal. Although the two were not strongly correlated, the nominal significance of the relationship also casts doubt on the potential causal relationship between these frailty exposure factors and cortical structure, and the clinical significance of the relationship is worth exploring. In particular, walk speed was associated with caudal anterior cingulate cortical thickness (without weighted); weight loss with lateral orbitofrontal surface area; weakness versus parahippocampal surface area compared to other non-positive results, and their association is worthy of further analysis. Notably, our results showed that the potential association between weakness and cortical structures was mainly focused on the effect on cortical SA, which accounted for about 3/4 of the total results, and the facilitating effect was overwhelming. Meanwhile, both walk speed and weakness contributed differentially to the increase of the superior temporal region, whereas weakness and physical activity showed opposite effects on the parahippocampal region (i.e: weakness decreased the parahippocampal surface area, and physical activity increased parahippocampal surface area). Thus, we are more skeptical that there is a currently undiscovered causal relationship between weakness and cortical structure than we are that these results are coincidental.

The development of brain structure is mainly regulated by genes, but the influence of acquired factors on brain structure is equally important. According to the radial unit hypothesis (RU hypothesis), the proliferation of neural progenitor cells drives the increase of cortical SA, while TH is determined by the number of divisions of neural cells (Rakic, 1988, 1995, 2009). In contrast, the diminished proliferation of neural progenitor cells during aging may be responsible for the changes in brain SA and TH, which are inextricably linked to frailty. According to Fjell et al. (2014) cortical reduction was found in most of the brain surfaces with age and the annual rate of change was around 0.5% in most of the regions, furthermore, the extent of annual changes in brain structures was greatest in frontal and temporal lobes, followed by significant changes observed in medial parietal regions. Kant et al. (2018) have found that frail compared to non-frail individuals The total brain volume as well as the gray matter volume was significantly lower in frail individuals and the cortical infarct area and the volume of WMH also showed an upward trend. Conservatively, frailty occurs with the aging process of the organism, and the brain SA and TH effects during aging may be related to the severity and time of occurrence of physical frailty.

Poor grip strength, as a crucial indicator for assessing physical function, is also a significant manifestation of the aging process and overall weakness of the body. Numerous studies have confirmed that low grip strength affects the cortical structure of the brain. A study on grip strength and brain structure revealed that stronger grip strength was associated with increased cortical structure in specific areas of the brain, including the ventral striatum, hippocampus, parahippocampal gyrus, temporal cortical areas, and pallidum (Jiang et al., 2022). According to our findings, weakness may affect the thickness of the caudal middle frontal, superior temporal, and rostral anterior cingulate, as well as the middle temporal, parahippocampal, and rostral middle frontal surface area. The middle temporal gyrus, on the other hand, is located in the hand knob region, and studies have shown that hand and arm activity activities this region (Willett et al., 2020). The effect of this on the middle temporal region is dramatic. Although there has been no reported evidence of a link between grip strength and the other results in this study, the impact of grip strength on the total cortical structure of the brain, as well as on subcortical areas, is significant. This is crucial for the precision and persuasiveness of our findings. Of course, because the brain has excellent remodeling properties, it is understandable that when the hand is exerted, the stimulation of the muscles and the nerves of the hand leads to physiological structural changes in the brain to adapt to changes in the environment.

Slow gait (walking speed) is inevitable for most older adults. Decreased walking speed has been associated with a variety of adverse outcomes, such as dementia and death (Verghese et al., 2007; Abellan van Kan et al., 2009). The results of the present study suggest that decreased walking speed in older adults is associated with an increase in the surface area of the inferior parietal lobe, caudal anterior cingulate gyrus, and superior temporal region. Since the inferior parietal and caudal anterior cingulate gyrus regions play a role in motor regulation (Paus, 2001; Claeys et al., 2003), it is understandable that walking speed would have an effect on these regions in frail older adults. In addition, a number of reliable studies have confirmed the effect of walking speed on cortical structures. Ezzati et al. (2015) showed that slower walking speeds were associated with the volume of the right cortex of the brain and the hippocampus (an important regulatory region for motor function). Other studies have also found a positive correlation between walking speed and the parietal and temporal lobes (Chen et al., 2020; Hupfeld et al., 2022). Therefore, it is reasonable to conclude that the stimulation of the inferior parietal lobe and caudate anterior cingulate gyrus regions by the inhomogeneity of walking speed, as brain regions involved in the regulation of locomotion, leads to adaptive changes in the brain in response to such variations. This leads to predictable changes in the inferior parietal, caudate, and anterior cingulate gyrus regions.

Weight loss occurs at various stages of a person’s life, but weight loss due to physical weakness differs from subjective weight loss (e.g., intentional weight loss). Therefore, weight loss induced by frailty can be considered pathological weight loss. In our findings, weight loss in the elderly may decrease the surface area of the lateral orbitofrontal cortex, increase the pericalcarine, and also increase the thickness of the inferior parietal lobe and the superior parietal lobule. As the body ages, the gradual weakening of its functions leads to a decreased demand for energy, resulting in a lower intake of food and ultimately weight loss. Studies have shown that the orbitofrontal cortex of the brain plays a key role in constructing value signals for food and other rewards. Analysis of functional MRI data indicates that food value represents a form of neural activity in the lateral orbitofrontal cortex (Suzuki et al., 2017). As the body’s appetite diminishes with age, the decreased assessment of food’s value results in the underutilization of the lateral orbitofrontal cortex region. The lack of function in this area may prompt other normally active regions to compensate, leading to a reduction in the surface area of the lateral orbitofrontal cortex. This phenomenon reflects the brain’s plasticity and remarkable adaptability. Unfortunately, this only pertains to the impact of diet-related weight loss on cortical structure, and the effects of other factors on cortical structure have not been reported yet.

Physical activity decreases with the onset of frailty, which is a significant characteristic of older adults experiencing frailty. Regular daily activity is beneficial for healthy aging and helps alleviate frailty (Kortebein et al., 2008; Theou et al., 2011). Studies have shown that a decrease in physical mobility leads to an expansion of the surface area of the parahippocampal and periaqueductal regions. Although the parahippocampal region plays a unique role in memory function (Aminoff et al., 2013), exercise has little to no effect on our memory perception. Interestingly, some studies have shown that physical activity induces a series of changes that enhance memory and cognitive functions (Loprinzi, 2019). Not only that, but some studies have revealed the effects of physical exercise on the parahippocampal region. Not only does actual exercise have an effect, but even imagined exercise affects the neural activity of the parahippocampal region and activates it during exercise (Jahn et al., 2004; la Fougère et al., 2010). Sun et al. (2024) conducted a magnetic resonance study on the relationship between physical exercise and the structure of the cerebral cortex. Researchers concluded that physical activity is linked to the thickness of the cerebral cortex. Although their results were different from those of the present study, this variance may be attributed to random error. We believe that this discrepancy is acceptable. However, it gives us reason to believe that there may be an undiscovered potential causal relationship between them.

Population aging has been increasing in all countries at present, and the prevalence of frailty has become inevitable. Even though research on frailty is accelerating, the coverage falls far short of expectations. The International Association of Gerontology Geriatrics (IAGG) defines frailty as a clinically significant syndrome, importantly characterized by both cognitive impairment and physical decline in the absence of dementia (Kelaiditi et al., 2013). In turn, the cerebral cortex is often recognized as the site of cognitive functioning (Filley and Fields, 2016). Therefore, it is urgent to address the link between frailty and brain structure either for physical health reasons or as a social public health issue. Both our study and previous research on cortical structures have shown that the development of frailty during the aging process is critical to the impact of cortical structures and that this impact threatens the quality of life and life expectancy of middle-aged and older adults. Therefore, we believe that a closer study of the mechanisms by which frailty affects the structural changes in the brain will enable us to monitor and prevent psychiatric disorders due to frailty in middle-aged and elderly people.

Although our study is a true reflection of the relationship between physical frailty and the cerebral cortex in older adults, there are still some limitations of the study. Firstly, all the people included in this study were from European populations and are not representative of the relationship between frailty and brain structure in other populations. Secondly, MRI data are derived from testing an all-age population, whereas our study only focused on middle-aged and older adults, so there is an unavoidable partial age interference between the exposure data and the resultant data. Thirdly, the analyzed study only reported a possible link between frailty and structural changes in the brain, and the exact mechanism remains to be investigated in a more recent study. Finally, the *P*-value for exposure exhaustion (*P* < 5 × 10^–5^) was too small, and the credibility as well as the stability of the results obtained were questionable, so it was discarded altogether.

Physical frailty, as a recognized clinical syndrome, is a potential causative factor for a variety of age-related diseases and a great challenge to global public health. Our study reveals a new factor affecting brain structure, but its direct association with disease remains to be studied in greater depth, and it is certain that it deserves to be emphasized and researched as a highly prevalent causative factor.

## 5 Conclusion

The results of our analyses suggest that there may be a causal relationship between physical weakness and multiple regions of cortical structure. However, the specific mechanisms of this effect remain to be investigated. The preliminary results of our analyses suggest that all indicators of physical frailty have varying degrees of influence on the structure of the cerebral cortex. Moreover, these relationships have been reported in the existing literature, indicating that it is both feasible and meaningful to examine the clinical significance of this association based on previous studies.

## Data availability statement

The original contributions presented in this study are included in this article/Supplementary material, further inquiries can be directed to the corresponding author.

## Ethics statement

The requirement of ethical approval was waived by the Dali University Ethics Committee for the studies involving humans because this study used public databases and these experimental data had already received ethical approval. The studies were conducted in accordance with the local legislation and institutional requirements. The ethics committee/institutional review board also waived the requirement of written informed consent for participation from the participants or the participants’ legal guardians because written informed consent for this study was submitted at the time of data collection. Written informed consent was obtained from the next of kin for the publication of any potentially identifiable images or data included in this article.

## Author contributions

XZ: Data curation, Investigation, Software, Writing – original draft, Writing – review and editing, Formal analysis, Resources, Methodology. ZW: Conceptualization, Software, Writing – review and editing. JZ: Formal analysis, Writing – review and editing. LZ: Data curation, Validation, Writing – review and editing. J-HN: Data curation, Formal analysis, Writing – review and editing. BJ: Writing – review and editing. YL: Supervision, Writing – review and editing. Y-ZZ: Funding acquisition, Investigation, Methodology, Writing – review and editing.

## Footnotes

1
http://www.nealelab.is/uk-biobank

2
https://enigmaconsortium.org/

3
www.phenoscanner.medschl
