# Supplementary material for: Association between physical frailty and cortical structure in middle-aged and elderly people: a Mendelian randomization study
Source: Front Aging Neurosci. 2024 May 22;16:1395553. doi: 10.3389/fnagi.2024.1395553 (PMC11150765; doi:10.3389/fnagi.2024.1395553)
Supplement: Supplementary file 1 [file Data_Sheet_1.docx]

**Effects of Key Exposure Factors of Physical Frailty on Cortical Structure in Middle-Aged and Older Adults: A Mendelian Randomization Study**

**
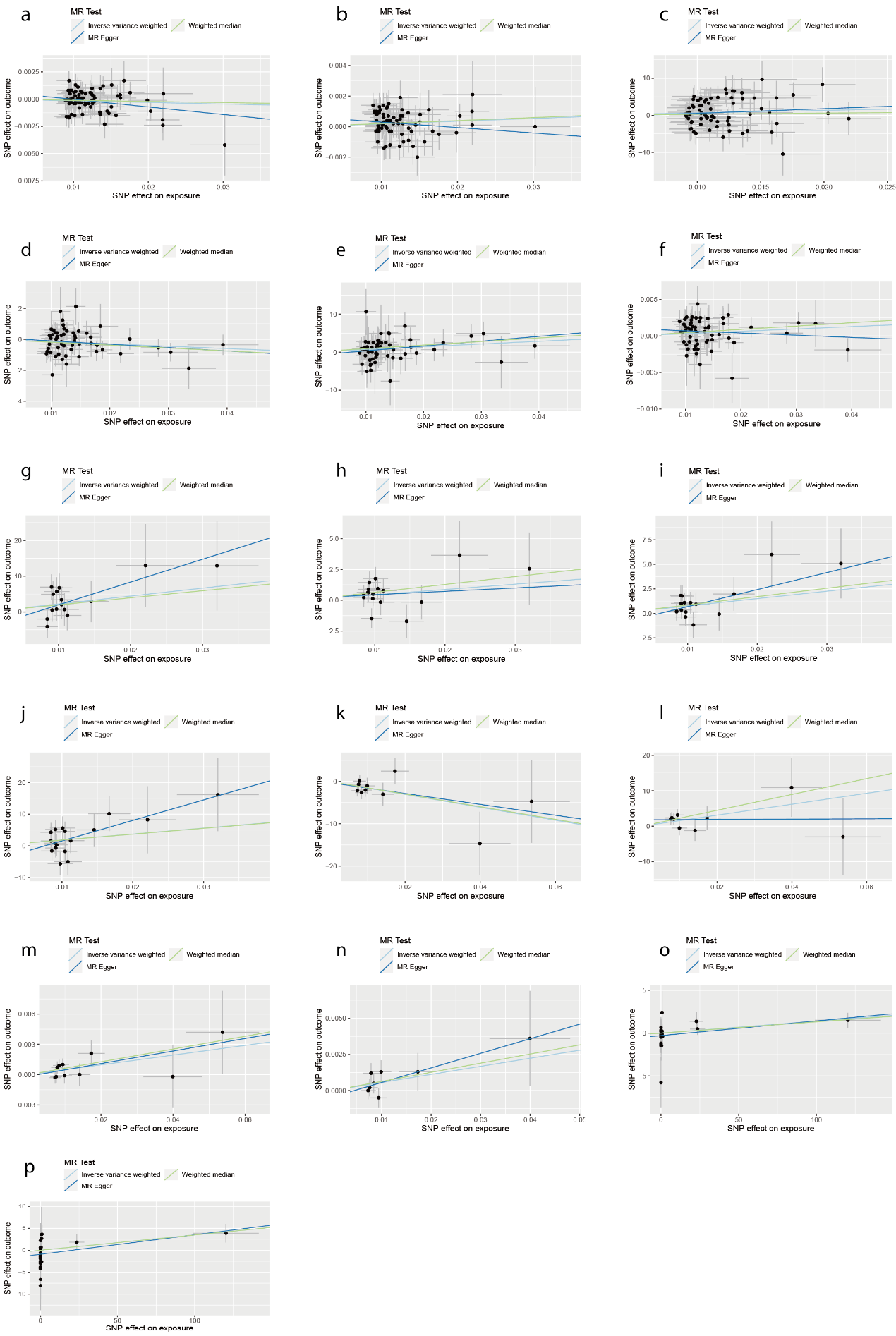
**

**Fig S1.** Scatter plots of nominal significant estimates from genetically predicted physical frailty on (a) weakness: with global weighted TH of caudal middle frontal; (b) weakness: with global weighted TH of superior temporal; (c) weakness: without global weighted SA of middle temporal; (d) weakness: with global weighted SA of parahippocampal; (e) weakness: with global weighted SA of rostral middle frontal; (f) TH of rostral anterior cingulate; (g) walk speed: with global weighted SA of inferior parietal; (h) walk speed: with global weighted SA of caudal anterior cingulate; (i) walk speed: without global weighted SA of caudal anterior cingulate; (j) walk speed: without global weighted SA of superior temporal;(k) weight loss: with global weighted SA of later orbitofrontal; (l) weight loss: with global weighted SA of pericalcarine; (m) weight loss: with global weighted TH of inferior parietal; (n) weight loss: with global weighted TH of superior parietal; (o) physical activity: without global weighted SA of parahippocampal; (p) physical activity: without global weighted SA of pericalcarine.


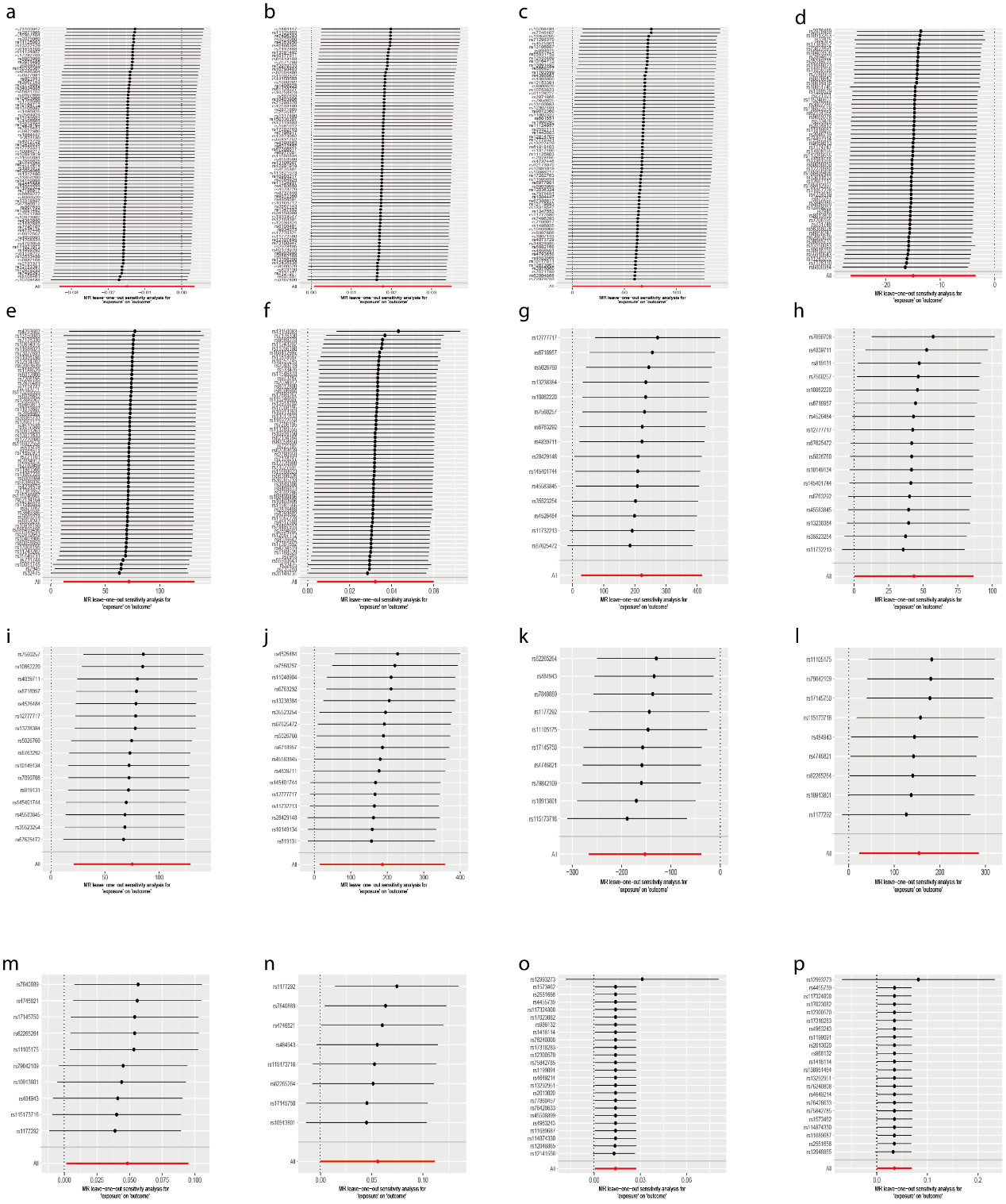


**Fig S2.** Leaveoneout of nominal significant estimates from genetically predicted physical frailty on (a) weakness: with global weighted TH of caudal middle frontal; (b) weakness: with global weighted TH of superior temporal; (c) weakness: without global weighted SA of middle temporal; (d) weakness: with global weighted SA of parahippocampal; (e) weakness: with global weighted SA of rostral middle frontal; (f) TH of rostral anterior cingulate; (g) walk speed: with global weighted SA of inferior parietal; (h) walk speed: with global weighted SA of caudal anterior cingulate; (i) walk speed: without global weighted SA of caudal anterior cingulate; (j) walk speed: without global weighted SA of superior temporal;(k) weight loss: with global weighted SA of later orbitofrontal; (l) weight loss: with global weighted SA of pericalcarine; (m) weight loss: with global weighted TH of inferior parietal; (n) weight loss: with global weighted TH of superior parietal; (o) physical activity: without global weighted SA of parahippocampal; (p) physical activity: without global weighted SA of pericalcarine.


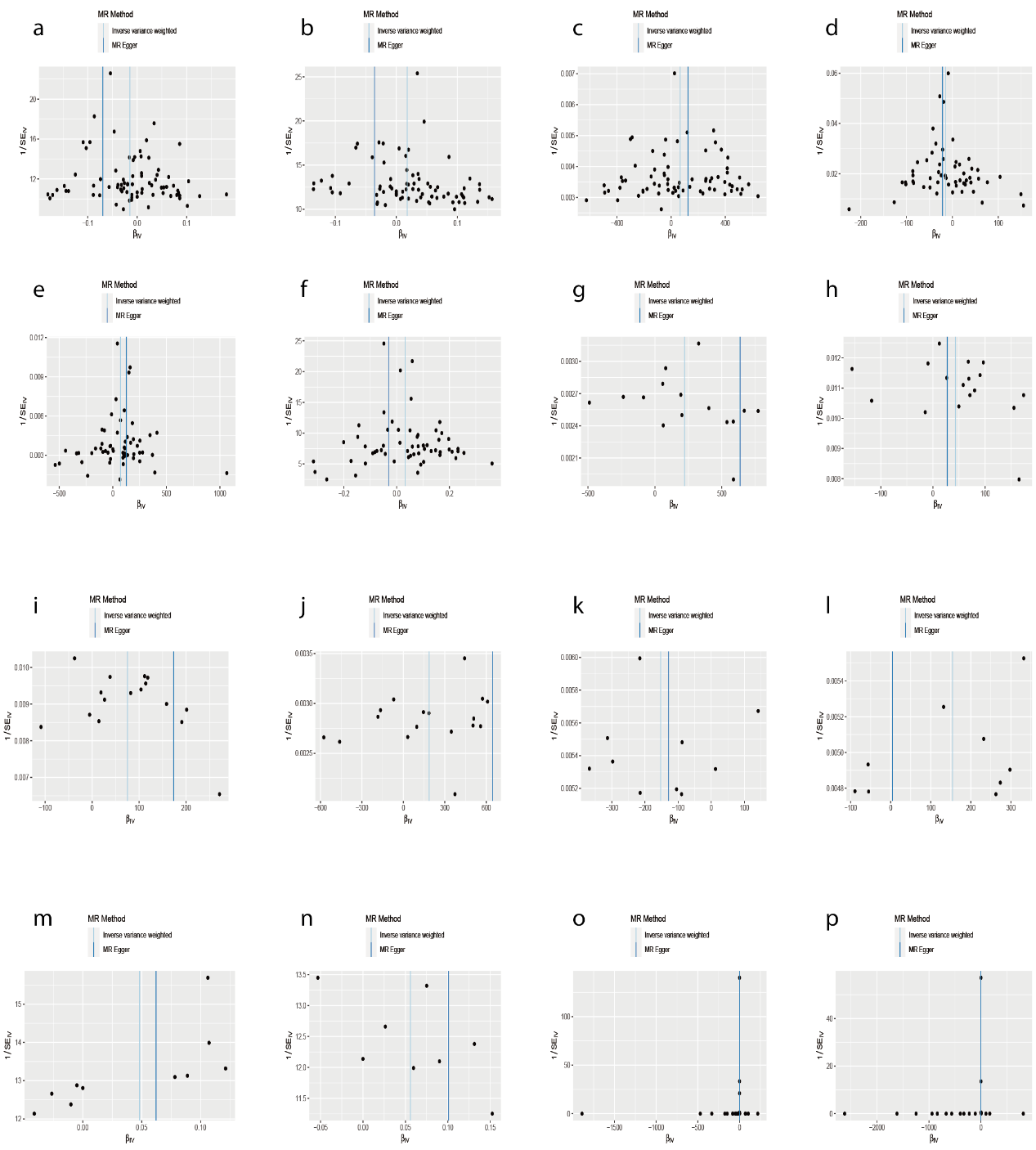


**Fig S3.** Funnel plots of nominal significant estimates from genetically predicted physical frailty on (a) weakness: with global weighted TH of caudal middle frontal; (b) weakness: with global weighted TH of superior temporal; (c) weakness: without global weighted SA of middle temporal; (d) weakness: with global weighted SA of parahippocampal; (e) weakness: with global weighted SA of rostral middle frontal; (f) TH of rostral anterior cingulate; (g) walk speed: with global weighted SA of inferior parietal; (h) walk speed: with global weighted SA of caudal anterior cingulate; (i) walk speed: without global weighted SA of caudal anterior cingulate; (j) walk speed: without global weighted SA of superior temporal;(k) weight loss: with global weighted SA of later orbitofrontal; (l) weight loss: with global weighted SA of pericalcarine; (m) weight loss: with global weighted TH of inferior parietal; (n) weight loss: with global weighted TH of superior parietal; (o) physical activity: without global weighted SA of parahippocampal; (p) physical activity: without global weighted SA of pericalcarine.
