# Supplementary material for: Association between physical frailty and cortical structure in middle-aged and elderly people: a Mendelian randomization study
Source: Front Aging Neurosci. 2024 May 22;16:1395553. doi: 10.3389/fnagi.2024.1395553 (PMC11150765; doi:10.3389/fnagi.2024.1395553)
Supplement: Supplementary file 2 [file Table_1.docx]

**Effects of Key Exposure Factors of Physical Frailty on Cortical Structure in Middle-Aged and Older Adults: A Mendelian Randomization Study**

Xin Zhang^1, +^, Zhen Wang^1, +^, Jing Zou^2^, Le Zhang^1^, Jing-Hua Ning^1^, Bei Jiang^3^, Yi Liang^4^ and Yu-Zhe Zhang^1^*

1. College of Basic Medical Sciences, Dali University, Dali, Yunnan, China

2. The First Affiliated Hospital of Dali University, Dali, Yunnan, China

3. Yunnan Key Laboratory of Screening and Research on Anti-pathogenic Plant Resources from West Yunnan (Cultivation), Dali, Yunnan, China

4. Princess Margaret Cancer Centre, TMDT-MaRS Centre, University Health Network, Toronto, ON, Canada

+. These authors contributed equally to this work

Definition of frailty used to match UK biobank GWAS data according to Fried's description

| Frailty indicators | UK biobank question |  |
| --- | --- | --- |
| Weakness | Grip strength was assessed using a calibrated J00105 hydraulic hand dynamometer (Lafayette Instrument Company, IN, USA) at mid-distance on the left and right arms, respectively. The average of the left and right arm measurements was used here, or the average available if measurements were missing for either arm.  Low grip strength cut-off values assessed according to gender and body mass index were taken from Fried (Fried, L. P., 2016):  Males:  BMI≤24 & grip strength≤29;  24.1≤BMI≤28 & grip strength≤30;  BMI>28 & grip strength≤32  Females:  BMI≤23 & grip strength≤17;  23.1≤BMI≤26 & grip strength≤17.3;  26.1≤BMI≤29 & grip strength≤18;  BMI>29 & grip strength≤21 |  |
| Exhaustion | Self-reported: “how often you felt tired or had low energy in the past two weeks?”  (response: more than half the days=1; nearly every day=1; not at all=0) |  |
| Weight loss | Self-reported: “Compared with one year ago, has your weight changed?”  (response: Yes, lost weight=1; No change or gain in weight=0) |  |
|  |  |  |
| Walk speed  Physical activity | Self-reported: “How would you describe your usual walking pace?”  (response: slow pace=1; brisk pace or steady average pace=0)  Self-reported: “In the last 4 weeks did you spend any time doing the following?”  (response: No physical activity or light DIY [eg: pruning, watering the lawn] in the past 4 weeks=1; Heavy DIY [eg: weeding, lawn mowing, carpentry, digging] or strenuous exercise=0) |  |

**Reference**

Fried, L. P., 2016. Investing in Health to Create a Third Demographic Dividend. Gerontologist 56 Suppl 2, S167-77.doi: 10.1093/geront/gnw035.
